# Mechanisms of Huhuang decoction in treating diabetic wounds: a network pharmacological and experimental study

**DOI:** 10.7150/ijms.108187

**Published:** 2025-03-10

**Authors:** Jie Zhang, Yan Shi, Jiaqiang Wang, Min Gao, Shan Zhong, Yunsheng Chen, Jiaqi Hao, Peilang Yang, Shun Xu, Yan Liu

**Affiliations:** 1 Department of Burn, Shanghai Burn Institute, Ruijin Hospital, Shanghai Jiao Tong University School of Medicine, Shanghai, China.; 2 Department of Burn and Plastic Surgery, Seventh People's Hospital Affiliated to Shanghai University of Traditional Chinese Medicine, Shanghai, China.

**Keywords:** Huhuang decoction, Diabetic wound healing, Network pharmacology, Vascular endothelial cells, Molecular docking

## Abstract

**Background:** Huhuang (HH) decoction, a composition of seven traditional Chinese medicines, has demonstrated clinical efficacy in wound healing. However, its pharmacological foundation and potential mechanisms remain unclear. This study aimed to elucidate the mechanisms of action of HH decoction in the treatment of diabetic wounds.

**Methods:** The chemical composition of HH decoction was analysed using ultra-high-performance liquid chromatography-quadrupole time-of-flight mass spectrometry. The targets of the HH decoction in treating diabetic wounds were predicted using network pharmacology. The gene ontology and pathway enrichment analyses were performed using the DAVID functional annotation tool. The compound targets and PPI networks were established using Cytoscape. Molecular docking was implemented using the AutoDock Vina software. Experimental verification was performed on the target prediction of the HH decoction in treating diabetic wounds, both *in vivo* and *in vitro*.

**Results:** The study identified 53 chemical components in HH decoction, with tetrahydropalmatine, emodin, rosmarinic acid, citric acid, berberine, and cryptotanshinone as key components for treating diabetic wounds. Twenty-one target genes were identified as core genes. Gene ontology analysis indicated that the therapeutic effects of HH on diabetic foot ulcers may occur through the regulation of cell proliferation, migration, and inflammation. Pathway enrichment was found to be mainly related to the HIF-1 and TNF signalling pathways. HH promoted proliferation, migration, and tube formation in vascular endothelial cells *in vitro*. Compared with the control group, the expression levels of HIF-1α, VEGF-α, cyclinD1 in the HH group were higher while the phosphorylation level of p65 in the HH group was significantly lower. The concentrations of IL-6, TNF-α, and IL-1β in wound tissue in the HH group were significantly lower than those in the control group. The expression levels of CD31, VEGF-α, Ki67 and HIF-1α in the wounds of diabetic rats in the HH group were higher than those in the control group.

**Conclusions:** The HH decoction promotes diabetic wound healing via multiple components, targets, and pathways. It may enhance vascular endothelial cell proliferation via cyclinD1, promote vascularization through the HIF-1α/VEGF-α signalling pathway, and inhibit inflammation through NF-κB signalling pathways.

## Introduction

The global prevalence of diabetic foot ulcers (DFUs) is 6.3% 1. DFUs represent one of the main complications of diabetes and are the primary cause of amputation in patients with diabetes. The main clinical manifestations of DFUs include re-epithelialisation dysfunction and prolonged wound healing. However, most current dressing scaffolds have shown poor therapeutic effects against DFUs 2. Traditional Chinese medicine (TCM) is gaining popularity for the treatment of DFUs because of its high efficacy, few side effects, and wide availability. TCM is a complex system that contains various active ingredients and targets multiple aspects, resulting in a synergistic therapeutic effect. Previous literature reports have indicated that TCM could promote diabetic wound healing by regulating signalling pathways, such as WNT, Notch, NF-κB, HIF-1α/VEGF, and TGF-β/Smad 3.

Huhuang (HH) decoction, developed by Seventh People's Hospital Affiliated to Shanghai University of Traditional Chinese Medicine, belongs to the TCM formula, which is characterised by comprehensiveness, synergistic effects, and minimal side effects. It has been confirmed in clinical practice that HH decoction could promote wound healing by topical application on wounds 4. However, the pharmacological foundations and mechanisms of the HH decoction in promoting wound healing have not yet been elucidated. HH decoction is composed of seven herbs: *Reynoutria japonica* Houtt (Chinese name Huzhang, 30 g), *Astragalus mongholicus* Bunge (Chinese name Huang qi, 15 g), *Phellodendron chinense* C.K.Schneid (Chinese name Huangbo, 10 g), *Coptis chinensis* Franch (Chinese name Huanglian, 10 g), *Salvia miltiorrhiza* Bunge (Chinese name Danshen, 10 g), *Paeonia veitchii* Lynch (Chinese name Chishao, 10 g), and *Spatholobus suberectus* Dunn (Chinese name Jixueteng, 15 g). Each component has been extensively studied and applied. *Reynoutria japonica* Houtt is a herb of the Polygonaceae family that is used for its anti-inflammatory, antioxidant, hepatoprotective, and neuroprotective properties 5. Specifically, *Reynoutria japonica* Houtt inhibits wound inflammation and promotes collagen synthesis, vascularization, epithelialisation, and wound healing 6. *Astragalus mongholicus* Bunge has demonstrated effectiveness in treating diabetes and its complications 7. APS2-1 extracted from *Astragalus mongholicus* Bunge promotes wound healing by inhibiting inflammation, regulating the cell cycle, and promoting the secretion of growth factors 8. *Phellodendron chinense* C.K.Schneid has anti-inflammatory, antibacterial, antioxidant, anti-tumour, hypoglycaemic, neuroprotective, and antidiarrheal properties. *Phellodendron chinense* C.K.Schneid promotes wound healing after anal fistulotomy 9. *Coptis chinensis* Franch extract is rich in alkaloids that can exert hypoglycaemic and antidiabetic effects 10. *Salvia miltiorrhiza* Bunge promotes blood circulation, removes blood stasis, and has demonstrated effectiveness in the treatment of diabetes and its complications 11. *Salvia miltiorrhiza* Bunge promotes vascularization in burn wounds 12. *Paeonia veitchii* Lynch can be used to promote diabetic wounds by activating the Nrf2 pathway 13. *Spatholobus suberectus* Dunn is a traditional blood-activating and stasis-dispelling medicine that promotes angiogenesis 14.

**Figure 1:**
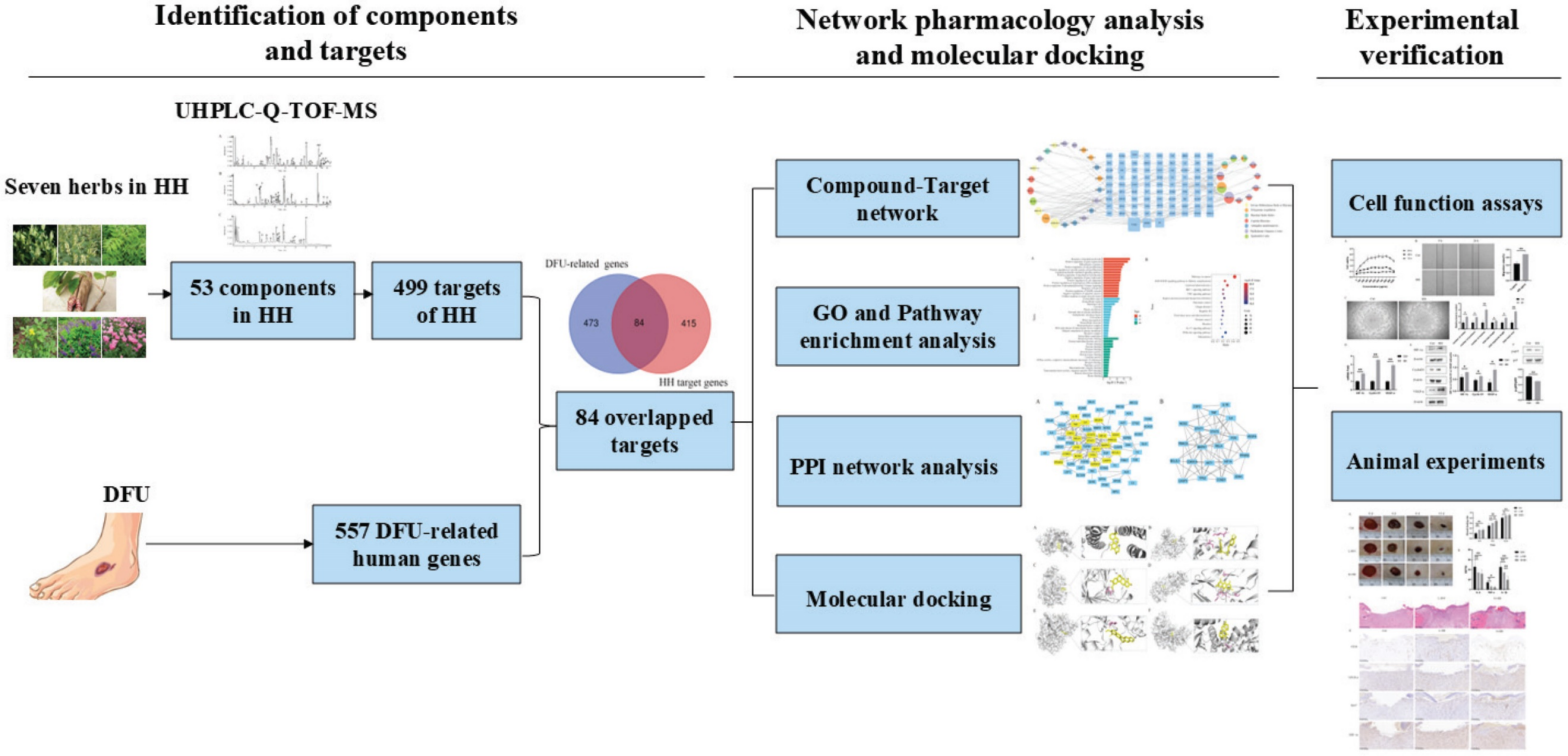
Strategy flowchart of investigating mechanisms of Huhuang decoction in treating diabetic wounds based on network pharmacology, molecular docking and experimental verification.

Network pharmacology was first proposed by Hopkins in 2007 and is based on network biology, bioinformatics, chemical informatics, and pharmacology. Its holistic view aligns perfectly with TCM principles 15. In this study, we employed network pharmacology to investigate the pharmacological network of HH decoction on diabetic wounds and predict the active “compound-target-pathway” network. We also verified the predicted mechanisms of action of HH decoction in diabetic wounds using both *in vivo* and *in vitro* experiments. This study provides valuable insights into the pharmacological foundations and mechanisms of HH decoction in promoting diabetic wound healing. These findings from our research would pave the way for novel and effective therapeutic strategies for the treatment of DFUs using TCM. The detailed design and strategy of the study are shown in Fig. 1.

## Materials and Methods

### Identification of main components of HH and network pharmacology analysis

#### Preparation of HH aqueous extract

The plant name has been checked with and in accordance with their respective accepted name in the World Flora Online (http://www.worldfloraonline.org/) database. To prepare the HH aqueous extract, the following traditional Chinese medicines were obtained: *Reynoutria japonica* Houtt (30 g, TS22C069-008), *Astragalus mongholicus* Bunge (15 g, TS22C069-009), *Phellodendron chinense* C.K.Schneid (10 g, TS22C069-010), *Coptis chinensis* Franch (10 g, TS22C069-011), *Salvia miltiorrhiza* Bunge (10 g, TS22C069-012), *Paeonia veitchii* Lynch (10 g, TS22C069-013), and *Spatholobus suberectus* Dunn (15 g, TS22C069-014). These components, totalling 100 g, were accurately weighed, mixed, and soaked in 1,000 mL of distilled water for 30 min. Subsequently, they were decocted for an additional 30 min. The solvent was filtered, and the supernatant was collected. The remaining TCM residue underwent the extraction process again. Finally, the supernatants were merged and then evaporated to dryness.

#### Phytochemical analysis of HH

Fingerprinting analysis was implemented using ultra-high-performance liquid chromatography-quadrupole time-of-flight mass spectrometry (UHPLC-Q-TOF-MS) to identify the chemical profile of the HH decoction. UHPLC-Q-TOF-MS was performed by Shanghai Standard Technology (Shanghai, China) using an ACQUITY UPLC H-Class PLUS (Waters Corporation, USA) and an AB Sciex Triple TOF® 4600 (SCIEX, USA).

#### Target prediction of HH components and collection of target genes for DFUs

The targets of HH components were extracted from TCMSP (https://tcmsp-e.com/tcmsp.php) 16, BATMAN-TCM (http://bionet.ncpsb.org.cn/batman-tcm/) 17 with a score cutoff >20, STITCH (http://stitch.embl.de/) 18 with a combined score ≥0.7, and Swiss Target Prediction (http://www.swisstargetprediction.ch/) 19 with a probability >0.5. DFUs-related human genes were collected from the Comparative Toxicogenomics database 20 (CTD; http://ctdbase.org/) with an inference score >15 and the GeneCards database 21 (http://www.genecards.org/) with a relevance score >15. Finally, the targets of the active ingredients of HH were mapped to DFUs-related human genes using Venn diagrams (http://bioinformatics.psb.ugent.be/webtools/Venn/).

#### Gene ontology (GO) and pathway enrichment analysis for DFUs-related targets of HH

The GO and pathway enrichment analyses were performed using the DAVID tool (https://david.ncifcrf.gov/) 22. Terms with thresholds of expression analysis systematic explorer (EASE) scores≤0.05 and Count≥2 were selected for further functional annotation clustering.

#### Construction of compound-target and PPI networks and analysis

To further characterise the therapeutic mechanism of HH loading on DFUs, a compound-target network was established using Cytoscape 3.9.1. Furthermore, the candidate targets were uploaded to the STRING database (https://string-db.org/) to obtain the PPI data with an interaction score ≥0.9. The PPI network was constructed using the Cytoscape software. The core gene network was screened based on six centrality measures provided by CytoNCA: betweenness centrality (BC), closeness centrality (CC), degree centrality (DC), eigenvector centrality (EC), local average connectivity-based method (LAC), and network centrality (NC). Targets with all six centrality measures above their medians were chosen as key targets of the PPI network.

#### Molecular docking studies

To assess the reliability of the predicted interactions between core components and targets, molecular docking was implemented using AutoDock Vina. The 2D structures of the core ligands were obtained from PubChem database (https://pubchem.ncbi.nlm.nih.gov/) and converted into 3D structures in MOL2 files using ChemBio3D. Crystal structures of the core target proteins were downloaded from the RCSB Protein Data Bank (https://www.rcsb.org/). The components and target proteins for docking were prepared using PyMOL and saved as PDBQT files using AutoDockTools. Finally, a docking simulation was implemented using AutoDock, and the lowest component-target binding affinity was chosen for further analysis. The action modes of the core components and their corresponding targets were analysed and visualised using the PyMOL software.

### Experimental validation

#### Cell culture

The human umbilical vein cell line EA.hy926 was acquired from FuHeng Biology (Shanghai, China). Cells were cultured in DMEM containing 10% foetal bovine serum, 100 mg/mL streptomycin, and 100 U/mL penicillin in a humidified incubator with 5% CO_2_ at 37 °C. The HH aqueous extract was redissolved in PBS to a concentration of 10 mg/mL and filtered with a 0.22 μm pore-size filter for cell treatment.

#### Cell viability assay

EA.hy926 (5,000 cells/100 μL) were seeded into each well of a 96-well plate. The cells in the control group were treated with PBS, and the cells in the HH group were treated with different concentrations of HH decoction (100-1,000 μg/mL) for 24 h or 48 h or 72 h. Five replicate wells were set up for each group. Then, the original medium was removed, and mixed solution (100 μL medium + 10 μL CCK-8(C0039, Beyotime, Shanghai, China)) was added to each well to incubate for 1 h. Finally, the absorbance was measured at 450 nm.

#### Migration assay

EA.hy926 (10^6^ cells/2 mL) were seeded into each well of a six-well plate. The cells in the control group were pre-treated with PBS, and the cells in the HH group were pre-treated with HH decoction (400 μg/mL) for 24 h. Scratch wounds were generated using a sterile 200 μL tip in each well when cells reached 100% confluence. The cells were gently rinsed twice with PBS. Photographs were taken at 0 and 24 h to record cell migration and were analysed using the ImageJ software.

#### Tube formation experiment

Fifty microliters (50 μL) of dissolved Corning Matrigel matrix (356234, Corning, USA) was incubated in each well of a 96-well plate at 37 °C for 30 min. The cells in the control group were pre-treated with PBS, and the cells in the HH group were pre-treated with HH decoction (400 μg/mL) for 24 h. Then, 3x10^4^ cells/100 μL in each group were seeded onto the Matrigel matrix in the 96-well plates and cultured for 6 h. Three replicate wells were set up for each group. The tube-like structures were photographed using a microscope. Quantitative analysis of tube formation was conducted using an angiogenesis analyser in ImageJ.

#### Western blotting

Equal amounts of total protein were resolved via 4-12% ExpressPlus™ PAGE Gel (M41215C, GenScript, USA) and transferred onto polyvinylidene fluoride (PVDF) membranes. The membranes were blocked using 5% non-fat milk in TBST and incubated with primary antibodies against HIF-1α (36169, CST), cyclinD1(2922, CST), VEGF-α (07-1420, Millipore), p-p65(3033, CST), p65(sc-8008, Santa Cruz) or β-actin (66009-1-Ig, Proteintech) at 1:1000 dilution overnight at 4 °C. The next day, the membranes were incubated with the appropriate secondary antibody at room temperature for 1 h. Finally, immunoreactive protein bands were detected with an enhanced chemiluminescence substrate kit, and band intensity was quantified using ImageJ.

#### Quantitative PCR

Total RNA was extracted using TRIzol reagent, and then reverse transcribed into cDNA using the HiScript® III RT SuperMix kit (R323-01, Vazyme). Quantitative PCR (qPCR) was implemented in triplicate using the ChamQ Universal SYBR qPCR Master Mix (Q711-02, Vazyme). The primers were synthesised by Shanghai Sangon Biotech (Shanghai, China) and presented as follows: HIF-1α forward GAACGTCGAAAAGAAAAGTCTCG and reverse CCTTATCAAGATGCGAACTCACA; cyclin D1 forward GCTGCGAAGTGGAAACCATC and reverse CCTCCTTCTGCACACATTTGAA; VEGF-α forward AGGGCAGAATCATCACGAAGT and reverse AGGGTCTCGATTGGATGGCA; GAPDH forward GGGAAACTGTGGCGTGAT and reverse GAGTGGGTGTCGCTGTTGA. The normalized gene expression was calculated via the 2^‑ΔΔCT^ method.

#### Animal experiments

Thirty SPF male SD rats weighing 120-150 g were provided and raised at the Animal Science Center of Ruijin Hospital. The experiment was conducted in conformity with the European Community guidelines (EEC Directive of 1986; 86/609/EEC) and was approved by the Animal Ethics Committee of Ruijin Hospital (RJ2024026). A diabetic rat model was established by intraperitoneal injection of streptozocin (60 mg/kg). Random blood sugar was tested once a week for eight consecutive weeks, and a value > 16.6 mM was considered an indicator of diabetes. The rats were randomly divided into control (n = 10), low dose HH (L-HH, n = 10) and high dose HH (H-HH, n = 10) groups. Four full-thickness defect wounds with diameters of 9 mm were created on the back of each rat. For the HH groups, 100 μL of L-HH decoction (50 mg/ml dissolved in PBS) or H-HH (100 mg/ml dissolved in PBS) was locally applied to the wounds every other day, while the control group received 100 μL of PBS applied locally to the wounds. After applying HH decoction or PBS, cover the wounds with 3M film. On the day of surgery (day 0) and on the 4th, 8th, and 12th days after injury, photographs were taken to record wound healing, and the wound and skin tissues within 5 mm of the wound margin were collected for histological detection. The wound area was calculated using the ImageJ software.

#### Histopathological examination and immunohistochemical staining

Histopathological examination was performed via haematoxylin and eosin staining. The vascularization of wound tissue was valued by immunohistochemical staining of CD31(ab182981, abcam) and VEGF-α. Cell proliferation was evaluated by immunohistochemical staining for Ki67 (ab15580, Abcam). Briefly, the paraffin sections were de-paraffinised and hydrated. After antigen retrieval and endogenous peroxidase blocking, paraffin sections were incubated with primary antibodies (CD31, VEGF-α, HIF-1α (Novus, NB100-105) or Ki67) overnight. The next day, the paraffin sections were washed with PBS and incubated with a secondary antibody conjugated to streptavidin-horseradish peroxidase. Paraffin sections were stained with DAB and counterstained with haematoxylin. The slices were photographed and recorded under a microscope.

#### ELISA

Wound tissues were ground with 9 times homogenate medium and then centrifuged for 10 min (4,000 r/min, 4 ℃) to obtain the supernatant. The protein concentration of the supernatant was measured using the bicinchoninic acid method. The concentrations of IL-6 (GER0001, Servicebio), tumour necrosis factor (TNF)-α (GER0004, Servicebio), and IL-1β (GER0002, Servicebio) in the wound tissues were determined according to the manufacturer's instructions. The measured protein concentrations were normalised to the total protein concentration in each sample.

#### Statistical analysis

All values were shown as means ± SD and analysed using GraphPad Prism 8. Differences between two groups were analysed using Student's *t*-test. One-way ANOVA or two-way ANOVA with Tukey's multiple comparisons test was used for three groups. *P* values < 0.05 were considered statistically significant.

## Results

### Network pharmacology analysis

#### Identification of components in HH

**Figure 2:**
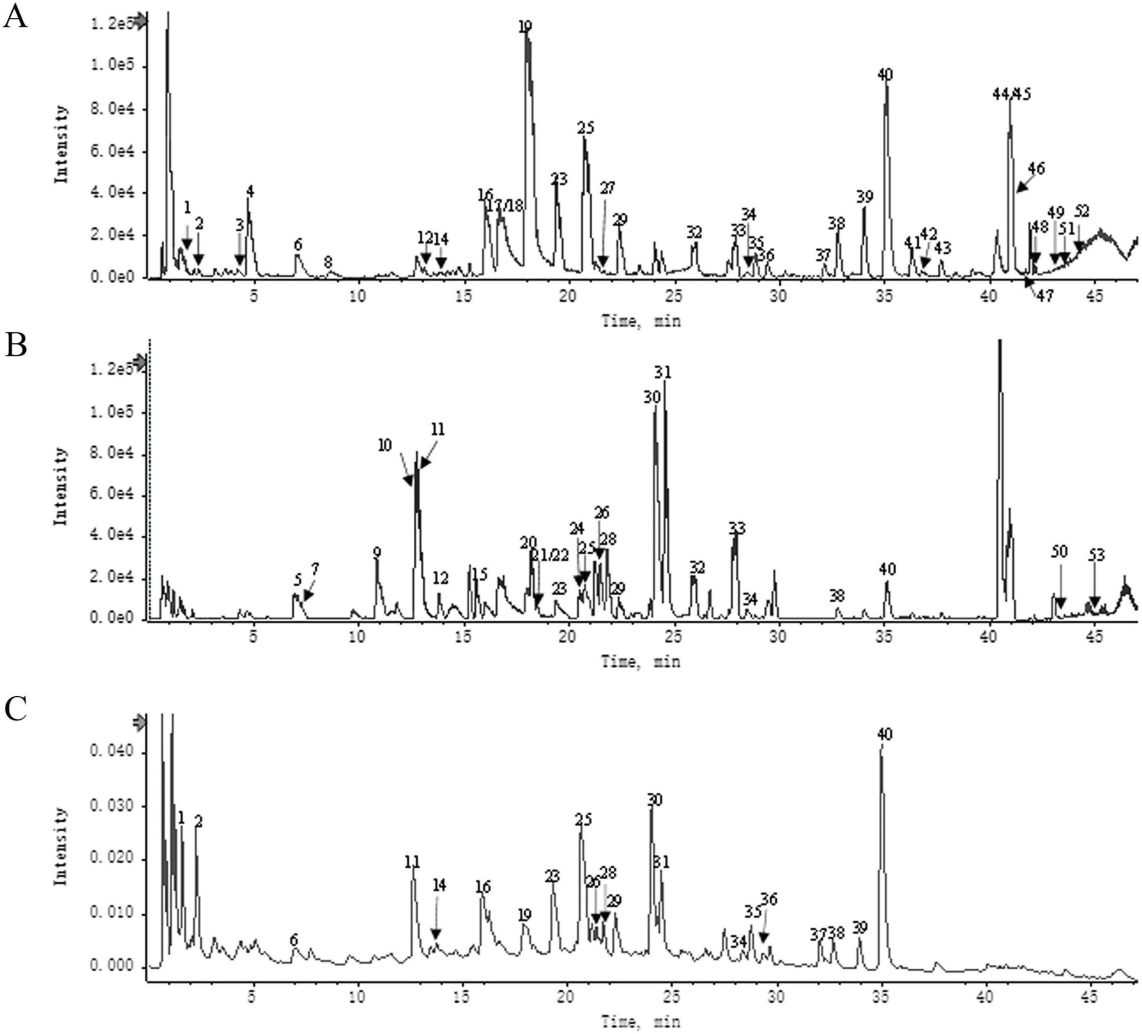
Components identification of HH decoction by UPLC-Q-TOF/MS. (A) UPLC-HRMS base peak ion current map (BPC) -negative ion mode of HH sample. (B) UPLC-HRMS base peak ion current map (BPC)-positive ion mode of HH sample. (C) UPLC UV chromatogram of HH sample-UV 254 nm.

A total of 53 main components of HH were identified using UHPLC-Q-TOF-MS (Fig. 2). Detailed information and structures of the 53 components are shown in Supplementary Table S1.

#### Target identification of HH on DFUs

**Figure 3:**
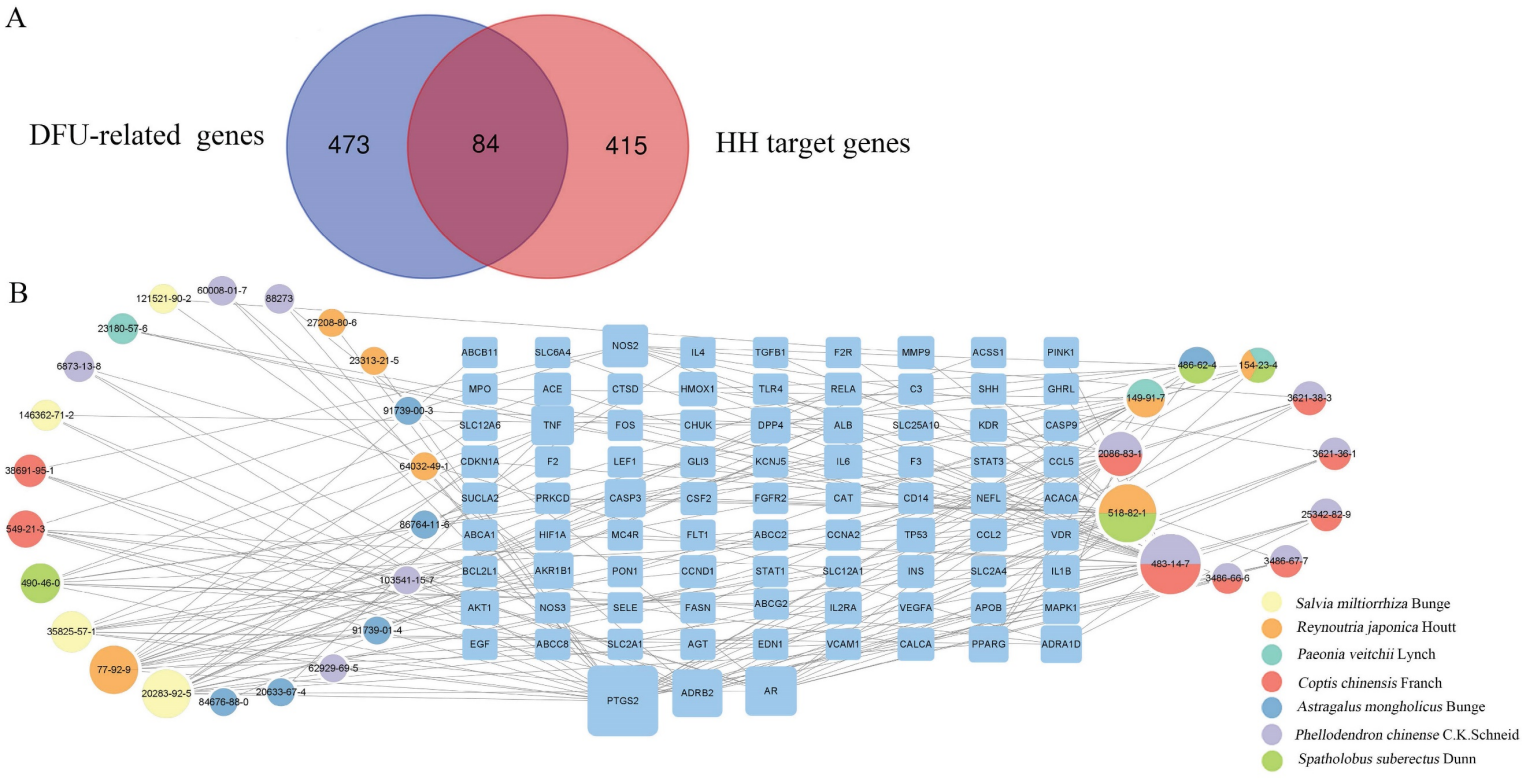
Identification of DFUs-related targets of HH and construction of compound-target network. (A) Intersection of DFUs-related genes and HH target genes. (B) Compound-target network of 84 overlapped genes.

According to preset filter conditions, 499 targets related to HH components were identified (Supplementary Table S2). In total, 557 DFUs-related human genes were identified from the CTD and GeneCards databases (Supplementary Table S3). These HH targets were mapped to DFUs-related human genes using Venn diagrams (Fig. 3A). As a result, 84 targets of the 33 components of HH were associated with DFUs (Supplementary Table S4).

#### Compound-target network and analysis

Various components of TCM formulae act on multiple targets to exert their pharmacological effects. To understand the therapeutic targets of HH in DFUs, a compound-target network was constructed (Fig. 3B), which included 117 nodes (33 for components in HH and 84 for potential overlapping targets). The degree values of all nodes in the network were calculated, and 11 components with degree values above the average were screened: tetrahydropalmatine (CAS 483-14-7, degree=23), emodin (CAS 518-82-1, degree=21), rosmarinic acid (CAS 20283-92-5, degree=15), citric acid (CAS 77-92-9, degree=15), berberine (CAS 2086-83-1, degree=12), cryptotanshinone (CAS 35825-57-1, degree=10), epicatechin (CAS 490-46-0, degree=9), gallic acid (CAS 149-91-7, degree=8), ononin (CAS 486-62-4, degree=7), oxyberberine (CAS 549-21-3, degree=7), and catechin (CAS 154-23-4, degree=6).

#### GO and pathway enrichment analysis

**Figure 4:**
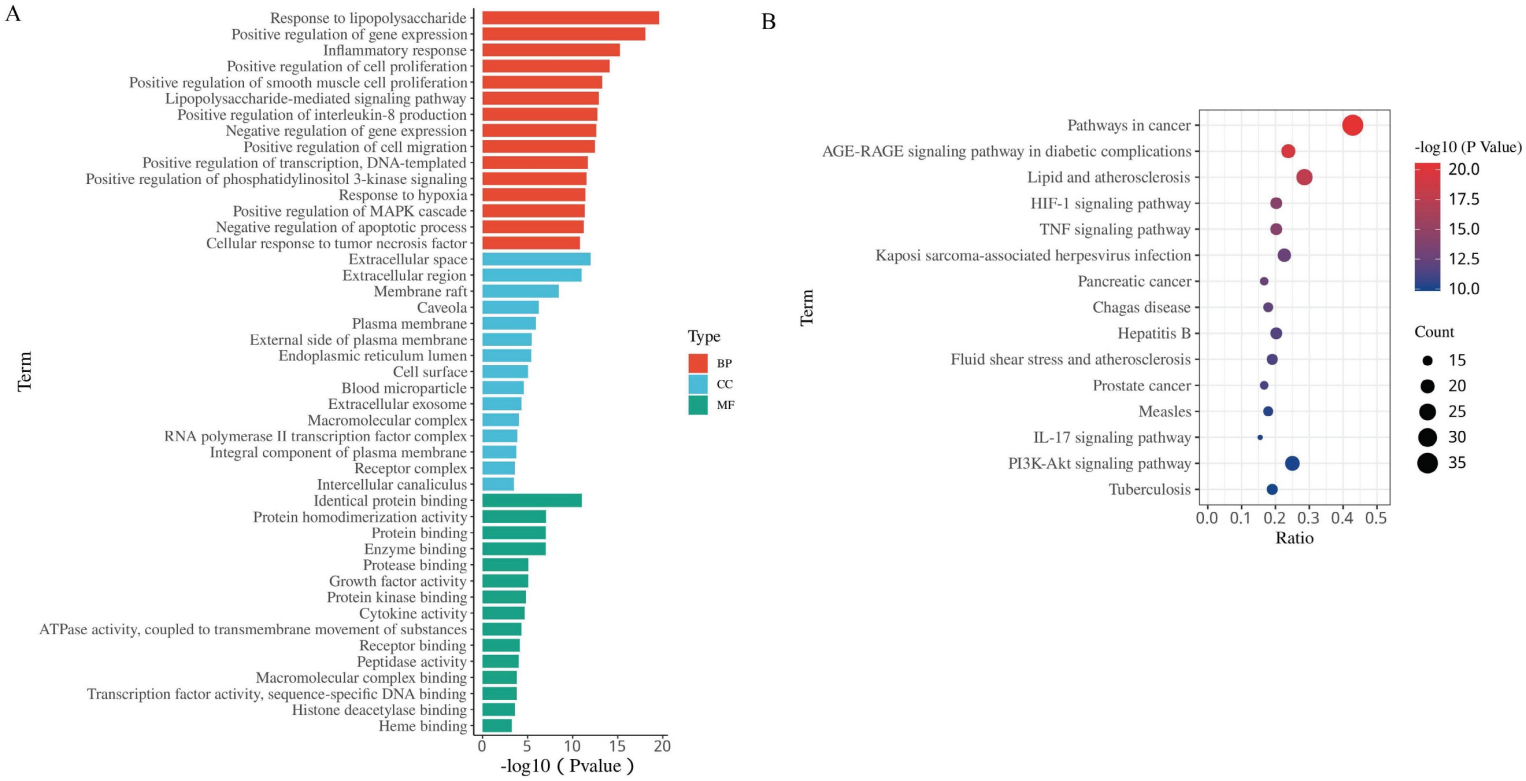
The 15 most significance of gene ontology (A) and KEGG pathway enrichment (B) analysis of therapy target genes of HH on DFUs.

To gain further insights into the biological functions of the 84 putative targets of HH in DFUs, GO and pathway enrichment analyses were fulfilled using the DAVID tool. There were 451 biological processes (BPs), 48 cellular components (CCs), and 52 molecular functions (MFs) according to the screening criteria of Count ≥2 and EASE scores ≤0.05 (Supplementary Table S5). The top 15 significantly enriched BPs, CCs, and MFs are shown in Fig. 4A. The results of GO analysis showed that the therapeutic effect of HH on DFUs might occur through the regulation of cell proliferation, migration, and inflammation. To explore the pathways underlying HH in DFUs, KEGG pathway analysis was conducted (Supplementary Table S6), and the top 15 significantly enriched pathways are shown in Fig. 4B. Among these, the AGE-RAGE signalling pathway in diabetic complications, HIF-1 signalling pathway, TNF signalling pathway, IL-17 signalling pathway, and PI3K-Akt signalling pathway are strongly involved in diabetic wounds.

#### PPI network analysis

**Figure 5:**
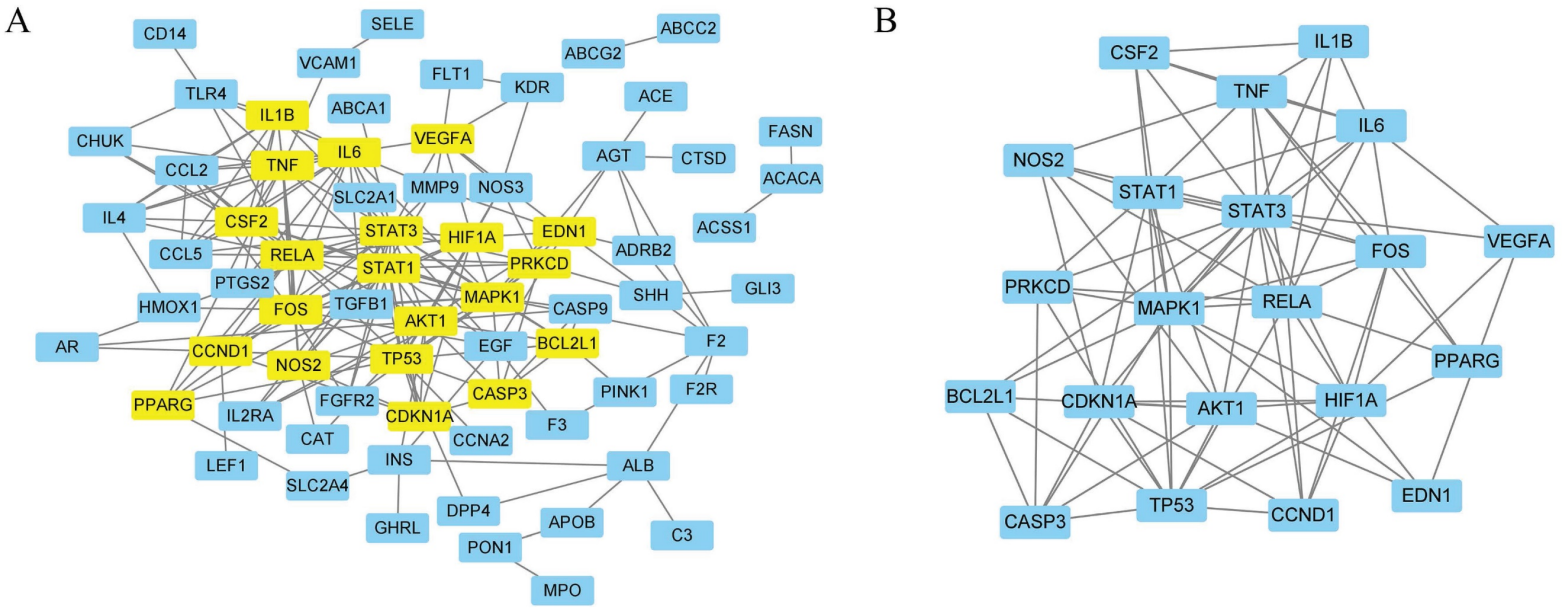
The PPI network of 84 overlapped targets (A) and key targets (B).

A total of 84 targets were uploaded to the STRING database, and disconnected nodes were removed. Finally, 70 targets with an interaction score ≥0.9 were screened to form the PPI network (Fig. 5A). Network analysis was performed using Cytoscape, and 21 genes were identified as core genes in the PPI network (Fig. 5B and Supplementary Table S7). The top 10 targets with the highest degrees were signal transducer activator of transcription 3 (STAT3), RELA proto-oncogene, NF-kB subunit (RELA), mitogen-activated protein kinase 1 (MAPK1), tumour protein p53 (TP53), signal transducer activator of transcription 1 (STAT1), hypoxia-inducible factor 1 subunit alpha (HIF1A), Fos proto-oncogene, AP-1 transcription factor subunit (FOS), interleukin 6 (IL6), AKT serine/threonine kinase 1 (AKT1), and TNF.

#### Docking results analysis

Molecular docking analysis between the 21 core genes and their corresponding HH components was performed using AutoDock Vina. A total of 27 pairs of molecular docking results were achieved (Table 1).

**Figure 6:**
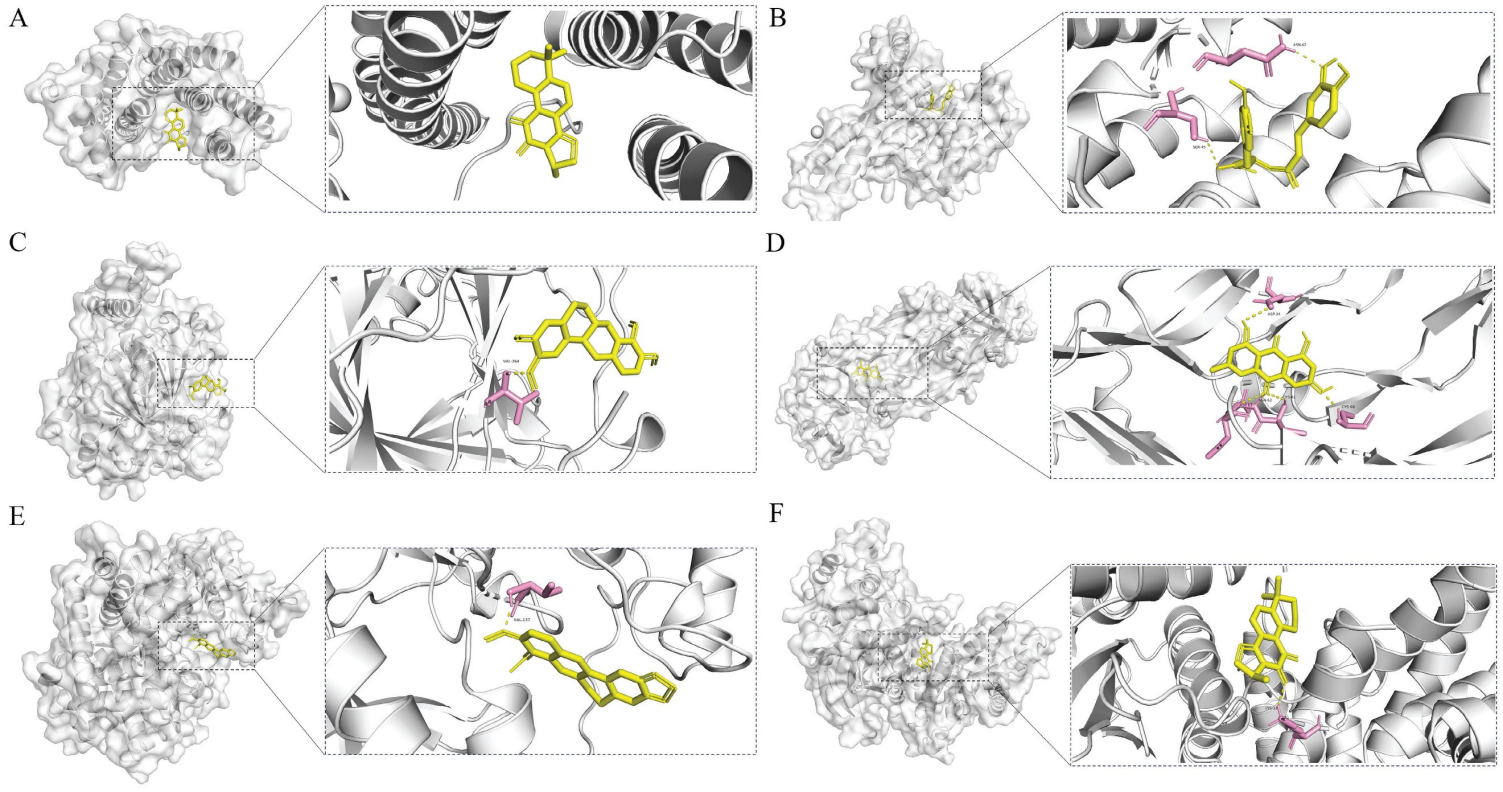
Schematic diagrams of the binding modes of representative component-targets. Cryptotanshinone with RELA (A); Rosmarinic acid with RELA (B); Tetrahydropalmatine with HIF1A (C); Emodin with VEGFA (D); Berberine with CCND1(E); Cryptotanshinone with CCND1 (F).

Affinity values were used to evaluate the molecular docking results; the lower the affinity value, the stronger the binding interaction. Generally, affinity values <-5 kcal/mol represent good binding activities, and affinity values <-7 kcal/mol represent even stronger binding activities between the component and target 23. In our study, all compound-target pairs exhibited affinity values of <-5 kcal/mol, and 23 pairs had an affinity of <-7 kcal/mol, indicating strong binding activities between the core components and targets. Network pharmacology analysis predicted that the therapeutic effects of HH on diabetic wounds were mainly related to vascular endothelial function, cell proliferation, and inflammation. Therefore, four core genes were selected (RELA, HIF1A, VEGFA, and CCND1), and the corresponding six pairs of molecular docking results are presented (Fig. 6).

### Experimental validation

#### HH decoction promoted proliferation, migration, and tube formation of vascular endothelial cells *in vitro*

**Figure 7:**
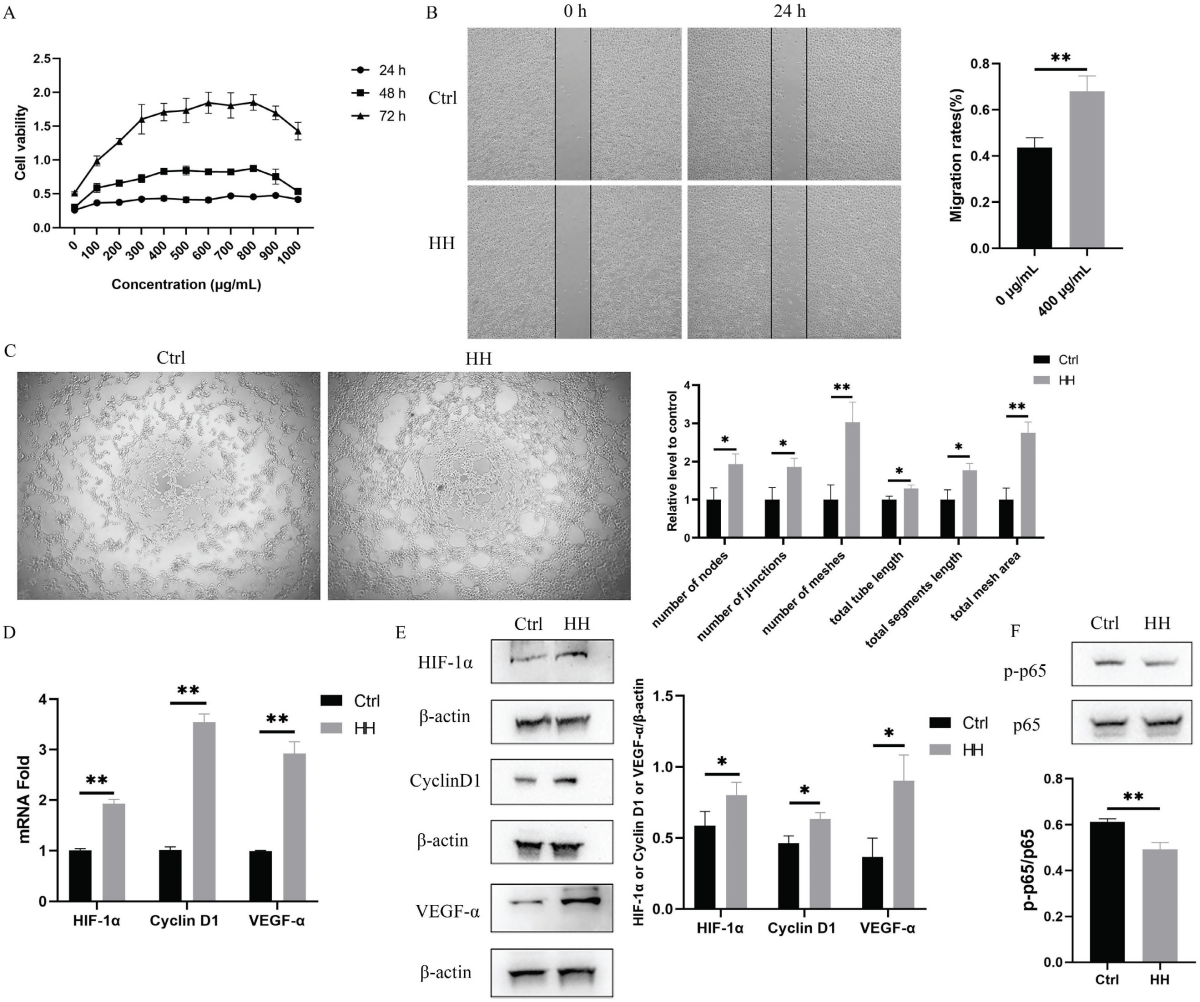
HH promoted proliferation, migration and tube formation of vascular endothelial cells *in vitro*. (A) Time (24 h, 48 h, 72 h) and dose (0-1000 μg/mL, with intervals of 100 μg/mL) dependent effects of HH treatment on the viability of EA.hy926 cells. (B) The representative images and statistical graphs of migration assay of EA.hy926 cells treated with or without HH (400 μg/mL) for 24 hours. (C) The representative images and statistical graphs of tube formation assay of EA.hy926 cells treated with or without HH (400 μg/mL) for 6 hours. (D) The normalized mRNA expressions of HIF-1α, CyclinD1 and VEGF-α of EA.hy926 cells treated with or without HH (400 μg/mL) for 24 hours. (E) Protein expressions of HIF-1α, CyclinD1 and VEGF-α of EA.hy926 cells treated with or without HH (400 μg/mL) for 24 hours. (F) Protein expressions of p-p65 and p65 of EA.hy926 cells treated with or without HH (400 μg/mL) for 24 hours. Data were presented as mean ± S.D. *P < 0.05, **P < 0.01.

To verify the biological effect of the HH decoction on vascular endothelial function, cell proliferation, migration, and tube formation were assessed using EA.hy926 cells. Cells were treated with different concentrations of HH (100-1,000 μg/mL) for various time points (24 h, 48 h, or 72 h) to observe the effect of HH on cell proliferation. As the concentration of the HH decoction increased, the promotion of cell proliferation became increasingly evident. However, higher concentrations did not necessarily result in better effects. At a concentration of 400 μg/mL, the proliferation reached a plateau period. When the concentration increased to 900 μg/mL, the proliferation effect weakened instead (Fig. 7A). Therefore, we selected the concentration of 400 μg/mL HH decoction for subsequent experimental verification. The area of cell migration in the HH group was significantly larger than that in the control group (Fig. 7B). Tube formation by cells in the HH group was also better than that in the control group. The six angiogenesis analyser-related indicators, including the number of nodes, junctions, meshes, total tube length, total segment length, and total mesh area, were significantly higher in the HH group than those in the control group (Fig. 7C).

In addition, we selected molecules associated to vascular endothelial function (HIF1A and VEGFA), proliferation (CCND1), and inflammation (RELA, also known as p65) among the core targets for verification. As shown in Fig. 7D and E, the expression levels of HIF-1α, VEGF-α, cyclinD1 in the HH group were higher than those in the control group. Additionally, the phosphorylation level of p65 decreased in cells treated with HH (Fig. 7F).

#### HH decoction accelerated diabetic wound healing *in vivo* by regulating proliferation, wound vascularization, and inflammation

**Figure 8:**
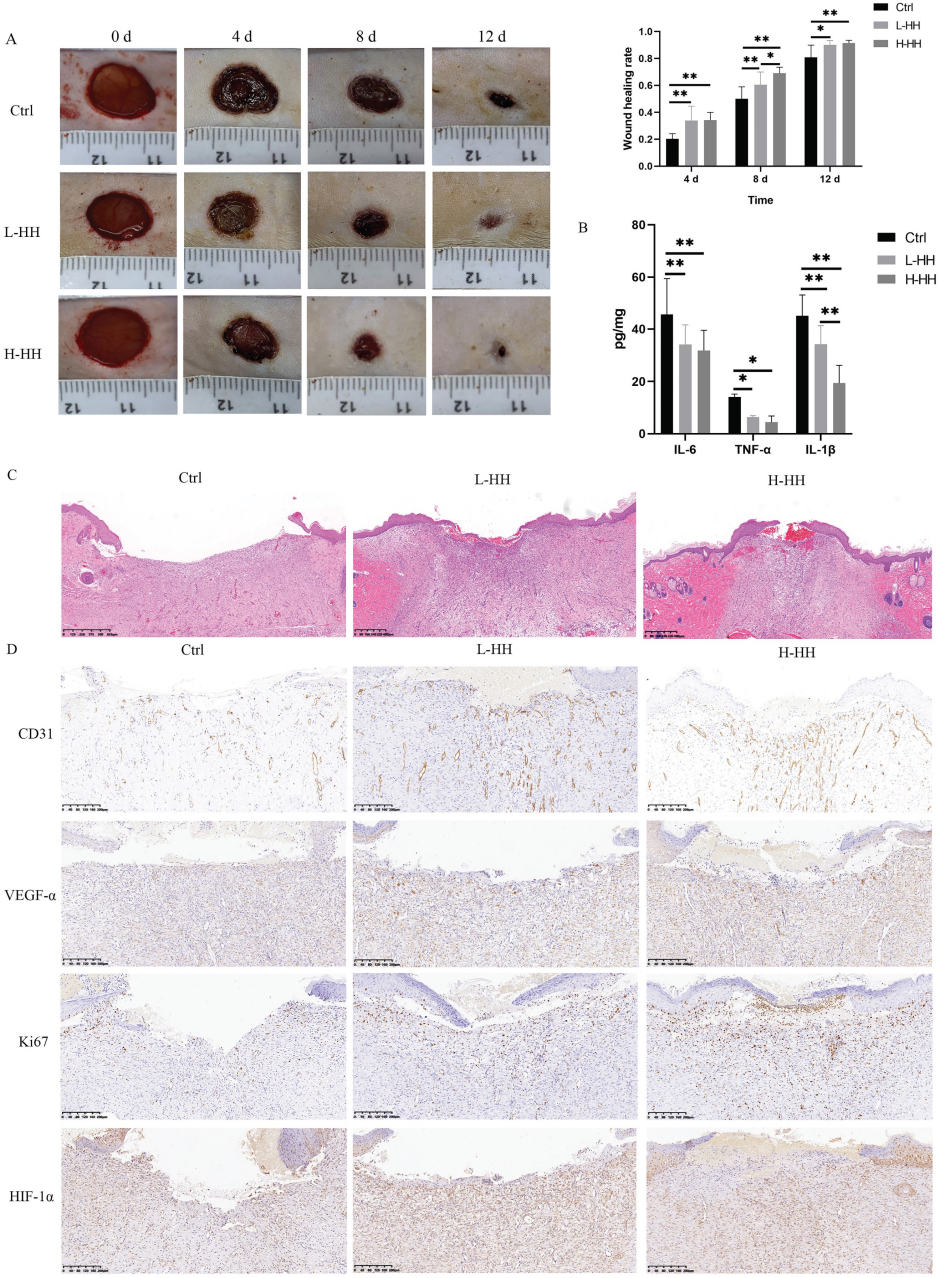
HH accelerated diabetic wound healing *in vivo* by regulating proliferation, wound vascularization and inflammation. (A) General photos of wound healing and quantitative statistics of wound healing rate. (B) The concentrations of IL-6, TNF-α and IL-1β in wound tissues (8 d) detected by ELISA. (C) Observation of epithelial migration tongue by HE staining (12 d). (D) Expressions of CD31 or VEGF-α or Ki67 or HIF-1α in wound tissues (12 d) detected by immunohistochemistry. Data were presented as mean ± S.D. *P < 0.05, **P < 0.01.

The wound-healing rates of diabetic rats in the L-HH or H-HH group were significantly higher than those in the control group at different time points. The wound-healing rates of diabetic rats in the H-HH group were higher than those in the L-HH group at different time points, but it was only statistically significant on the eighth day (Fig. 8A). The concentrations of IL-6, TNF-α, and IL-1β in wound tissue in the HH groups were significantly lower than those in the control group (Fig. 8B). Compared with the control group, the epithelial migration of the tongue in the HH goups increased in a concentration-dependent manner (Fig. 8C). The expression levels of CD31, VEGF-α, Ki67, and HIF-1α in the wounds of diabetic rats in the HH groups were higher than those in the control group in a concentration-dependent manner. (Fig. 8D).

## Discussion

TCM relies on various compounds to exert pharmacological effects by targeting multiple pathways, making it a potentially valuable approach for treating DFUs. However, the complexity of TCM composition poses challenges in understanding its exact pharmacological mechanisms. In the current study, we used a network pharmacology approach to elucidate the mechanism by which HH decoction alleviates DFUs.

The complexity of TCM often centres around specific key components that contribute to its efficacy. In this study, we identified 53 main components of HH using UHPLC-Q-TOF-MS. From the compound-target network analysis, we highlighted six core components with the highest degree values: tetrahydropalmatine, emodin, rosmarinic acid, citric acid, berberine, and cryptotanshinone. These compounds are likely responsible for the major therapeutic effects of HH against DFUs. Tetrahydropalmatine promotes wound healing by modulating angiogenesis 24. A previous study showed that tetrahydropalmatine activated the PI3K/Akt/eNOS/NO pathway and upregulated the expression of HIF-1α and VEGF 25. Tetrahydropalmatine also exerts anti-inflammatory effects, which are closely related to the P2X3/TRPV1, TRAF6/JNK, PI3K/Akt/eNOS/NO and NF-κB signaling pathways 26. Emodin has been shown to accelerate diabetic wound healing by promoting the polarisation of anti-inflammatory macrophages 27. Rosmarinic acid exhibits antioxidant, anti-inflammatory, and photoprotective functions 28. Rosmarinic acid can be used to treat osteoarthritis by inhibiting p65 nuclear translocation 29. Moreover, a novel nanofibre material containing rosmarinic acid has been reported to promote wound healing 30. Citric acid has been reported as an antibacterial agent for the treatment of diabetic wounds 31 and can promote the healing of chronic leprosy wounds 32. A study has reported that bioactive poly(salicylic acid)-poly(citric acid) scaffolds can promote the healing of diabetic wounds through anti-inflammatory, antioxidant, and pro-angiogenic effects 33. Several studies have reported that berberine promotes wound healing 34-36. Cryptotanshinone, an active component of *Salviae Miltiorrhizae* Bunge, inhibits inflammation via the TLR4-MyD88/NF-κB/MAPK pathway 37. Cryptotanshinone can promote diabetic wound healing by regulating inflammation, angiogenesis and extracellular matrix remodeling 38. These six compounds have potential synergistic effects with each other, collectively promoting wound healing. These six components all possess anti-inflammatory and antioxidant effects. Tetrahydropalmatine, citric acid and cryptotanshinone could promote vascularization. Taken together, these studies and our experimental results support the notion that network prediction is an effective method for unravelling the network pharmacological mechanisms of HH.

GO enrichment analysis revealed that the molecular mechanisms of HH in DFUs primarily involve cell proliferation, migration, and inflammation. KEGG pathway enrichment analysis indicated that the therapeutic effect of HH on DFUs involved the AGE-RAGE, HIF-1, TNF, IL-17, and PI3K-Akt pathways. These signalling pathways are consistent with previously reported pathways that play significant roles in the progression of DFUs 39.

In addition, 21 core target genes of HH on DFUs were identified, and these targets were primarily related to inflammation (RELA, TNF, IL6, IL1B, CSF2, and PPARG), vascular endothelial function (HIF1A, VEGFA, NOS2, and EDN1), cell survival, migration, and proliferation (CCND1, CDKN1A, STAT3, STAT1, FOS, MAPK1, AKT1, PRKCD, TP53, BCL2L1, and CASP3). Molecular docking was used to further elucidate the binding modes between the core components and targets. The results showed that all core components-targets have a good binding affinity (<-5 kcal/mol), which further confirmed the reliability of network pharmacology predictions.

Through the analysis of the above core genes, we speculated that the mechanism of HH in treating diabetic wounds mainly involves vascular endothelial function, proliferation, and inflammation. Furthermore, impaired vascularization is a critical factor in diabetic wound healing. Therefore, in this study, we employed vascular endothelial cells for *in vitro* experiments to specifically investigate the vascularization effects of HH decoction. We selected vascular function-related proteins (HIF-1α, VEGF-α), proliferation-related proteins (cyclinD1), and inflammation-related proteins (p65, IL-6, TNF-α and IL-1β) from 21 core targets to further evaluate the molecular mechanisms. *In vitro* experiments using EA.hy926 cells and *in vivo* experiments on diabetic rat wounds were conducted to assess the therapeutic effects of HH. Endothelial dysfunction and inhibited angiogenesis due to high glucose levels can hinder wound healing 40. As a result, the effect of HH on vascular endothelial function was verified using migration and tube formation experiments. The *in vitro* results showed that HH promoted the migration and tube formation of vascular endothelial cells. The *in vivo* results showed that the expression of the vascular marker CD31 increased in wound tissue treated with HH. Both *in vitro* and *in vivo* results showed that the expression of pro-angiogenic factors (HIF-1α and VEGF-α) increased after HH treatment. These results suggest that HH promotes vascularization in diabetic wounds via the HIF-1α/VEGF-α pathway. In addition, HH also promoted proliferation. The cell proliferation and the expression of cyclinD1 in the HH group were increased. The expression of ki67, a marker of cell proliferation, was increased in HH-treated wounds *in vivo*. Sustained inflammation mediated by NF-κB signalling pathway activation is an important feature of diabetic wounds 41. Elevated expression levels of p-p65 imply activation of the NF-κB signalling pathway 42. We detected the activation level of the NF-κB p65 signalling pathway in EA.hy926 cells and the expression of pro-inflammatory cytokines (IL-6, TNF-α, and IL-1β) in wound tissues. The results showed that HH decoction inhibited the activation of p-p65 in endothelial cells and reduced the expression of proinflammatory cytokines in diabetic wounds. In this study, we focused on the intracellular cytokines by analyzing lysates of wound tissues, as these cytokines are directly involved in the local inflammatory response and wound healing process. Our future research will measure cytokine levels in blood to help us better understand the overall effects of HH decoction. Although endothelial cells can secrete some inflammatory factors, their primary source is still macrophages. Future research should evaluate the effects of HH decoction on cell behavior and cytokine secretion of macrophages.

In summary, the HH decoction is a promising comprehensive formulation that regulates the above therapeutic targets to promote diabetic wound healing. To the best of our knowledge, this is the first study to comprehensively screen and validate therapeutic targets for HH decoction in diabetic wounds. However, there are some limitations in our current research. First, the current experiments were conducted in animal models, which may not fully replicate the complexity of human diabetic wounds. Second, the precise contributions of individual components in HH decoction to the overall therapeutic effect require further investigation. Third, we only focused on the effects of HH decoction on endothelial cells and not on fibroblasts or keratinocytes or macrophages. Future research should investigate its effects on other wound-repair cells to understand its broader therapeutic potential.

## Conclusion

In summary, this study explored the mechanisms by which the HH decoction promotes diabetic wound healing based on network pharmacology, molecular docking, and experimental verification. HH decoction enhances vascular endothelial cell proliferation via cyclinD1, promotes vascularization through the HIF-1α/VEGF-α signalling pathway, and inhibits inflammation through the NF-κB signalling pathways. These findings reveal the potential mechanisms of action of HH decoction in diabetic wounds treatment and provide a theoretical foundation for its clinical use. Given its natural composition and efficacy, HH decoction could become a cost-effective therapeutic option for diabetic patients, especially in resource-limited settings. Future clinical trials are needed to validate its safety and efficacy, potentially integrating it into standard wound care protocols.

## Supplementary Material

Supplementary tables.

## Figures and Tables

**Table 1:** Docking scores between core components and targets

Core targets	PDB ID	HH components	Affinity (kcal/mol)
FOS	1FOS	tetrahydropalmatine	-8.1
IL1B	1HIB	tetrahydropalmatine	-6.8
HIF1A	1h2k	tetrahydropalmatine	-6.9
IL6	1ALU	tetrahydropalmatine	-6.1
CDKN1A	2ZVV	rosmarinic acid	-7.1
CASP3	3H0E	rosmarinic acid	-8.2
		berberine	-8.9
RELA	6NV2	rosmarinic acid	-7.4
		cryptotanshinone	-8.3
STAT1	1YVL	rosmarinic acid	-7.7
PPARG	3E00	rosmarinic acid	-8.2
MAPK1	1TVO	rosmarinic acid	-7.9
		berberine	-8.4
AKT1	4EJN	citric acid	-5.3
		berberine	-10.6
CCND1	2W96	berberine	-7.5
		cryptotanshinone	-8.4
TP53	3ZME	berberine	-8.4
NOS2	4NOS	berberine	-8.3
BCL2L1	1R2E	cryptotanshinone	-7.9
EDN1	6DK5	cryptotanshinone	-8.5
STAT3	1BG1	cryptotanshinone	-8.2
TNF	2AZ5	cryptotanshinone	-8.5
VEGFA	4QAF	Emodin	-7.4
CSF2	1CSG	Emodin	-7.7
		Catechin	-7.1
PRKCD	1YRK	Emodin	-7.2

## Abbreviations

HH: Huhuang
UHPLC-Q-TOF-MS: Ultra-high-performance liquid chromatography-quadrupole time-of-flight mass spectrometry
DAVID: The Database for Annotation, Visualization and Integrated Discovery
TNF-α: Tumour necrosis factor alpha
HIF-1α: Hypoxia inducible factor 1 alpha
VEGF-α: Vascular endothelial growth factor alpha
IL-6: Interleukin 6
IL-1β: Interleukin 1 beta
NF-κB: Nuclear factor kappa B
DFUs: Diabetic foot ulcers
TCM: Traditional Chinese medicine
GO: Gene ontology
PPI: Protein-protein interaction
