# Supplementary material for: Mechanisms of Huhuang decoction in treating diabetic wounds: a network pharmacological and experimental study
Source: Int J Med Sci. 2025 Mar 10;22(8):1811–24. doi: 10.7150/ijms.108187 (PMC11983308; doi:10.7150/ijms.108187)
Supplement: Supplementary file 1 — Supplementary tables. [file ijmsv22p1811s1.zip › supplementary files/Supplementary Table S1.DOCX]

**Table S1 |** A list of the 53 main components of HH lotion and their corresponding structures.

| **No.** | **Molecular Formula** | **CAS** | **Molecule Name** | **Structure** | **Herb** |
| --- | --- | --- | --- | --- | --- |
| 1 | C_6_H_8_O_7_ | 77-92-9 | Citric acid | 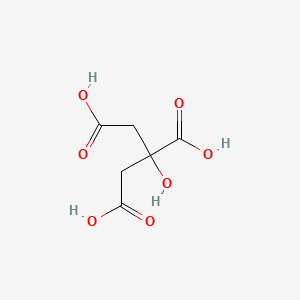 | *Reynoutria japonica* Houtt |
| 2 | C_7_H_6_O_5_ | 149-91-7 | Gallic acid | 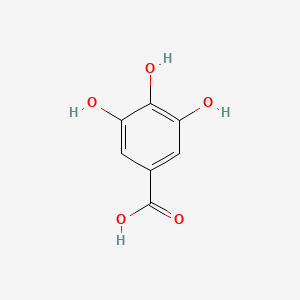 | *Paeonia veitchii* Lynch*, Reynoutria japonica* Houtt |
| 3 | C_15_H_20_O_9_ | 2585654-76-6 | 9-O-glucosyl 4-hydroxyphenyl-actic acid | 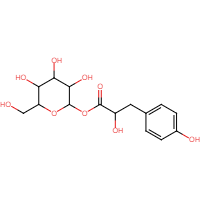 | *Salvia miltiorrhiza* Bunge |
| 4 | C_15_H_20_O_10_ | 2585654-77-7 | Danshensu 7-O-glucoside | 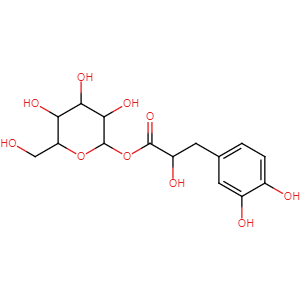 | *Salvia miltiorrhiza* Bunge |
| 5 | C_18_H_19_NO_3_ | 103541-15-7 | Clausenamide | 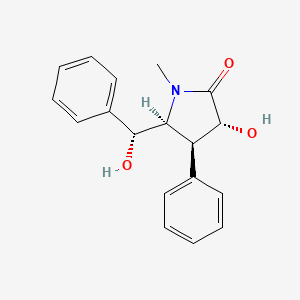 | *Phellodendron chinense* C.K.Schneid |
| 6 | C_11_H_12_O_7_ | 469-65-8 | Piscidic Acid | 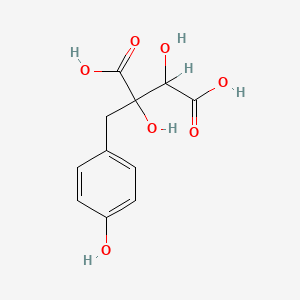 | *Spatholobus suberectus* Dunn |
| 7 | C_19_H_24_NO_3_^+^ | 6801-40-7 | Magnocurarine | 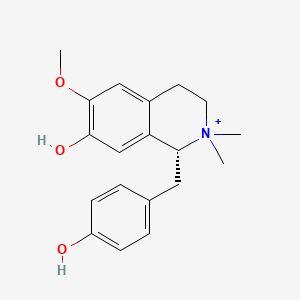 | *Phellodendron chinense* C.K.Schneid |
| 8 | C_13_H_16_O_9_ | NA | Protocatechuic acid 3-glucoside | 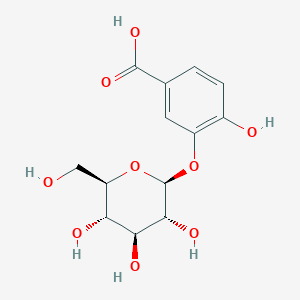 | *Spatholobus suberectus* Dunn |
| 9 | C_20_H_24_NO_4_^+^ | 6873-13-8 | Phellodendrine | 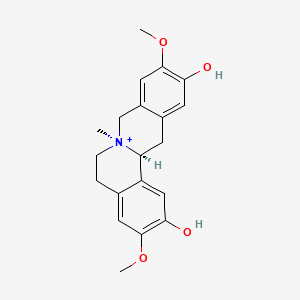 | *Phellodendron chinense* C.K.Schneid |
| 10 | C_23_H_29_NO_8_ | NA | N-Methylhigenamine 7-glucopyranoside | 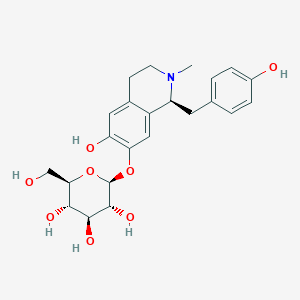 | *Phellodendron chinense* C.K.Schneid |
| 11 | C_20_H_24_NO_4_^+^ | 2141-09-5 | Magnoflorine | 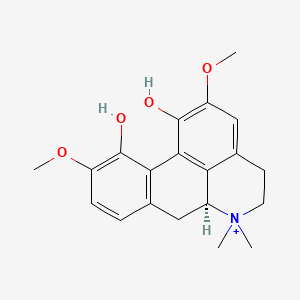 | *Phellodendron chinense* C.K.Schneid |
| 12 | C_15_H_14_O_6_ | 154-23-4 | Catechin | 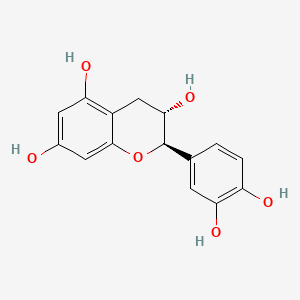 | *Spatholobus suberectus* Dunn*,Paeonia veitchii* Lynch*,*  *Reynoutria japonica* Houtt |
| 13 | C_19_H_24_NO_3_^+^ | 60008-01-7 | Oblongine | 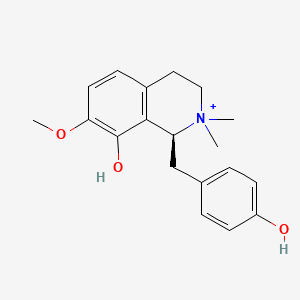 | *Phellodendron chinense* C.K.Schneid |
| 14 | C_23_H_28_O_12_ | 1161828-55-2 | Oxyalbiflorin | 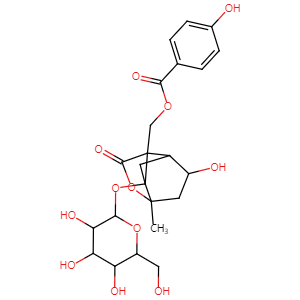 | *Paeonia veitchii* Lynch |
| 15 | C_21_H_26_NO_4_^+^ | 25342-82-9 | Menisperine | 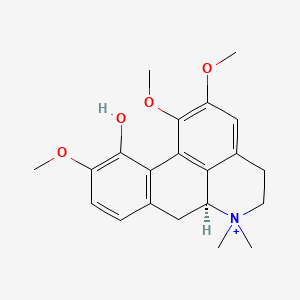 | *Phellodendron chinense* C.K.Schneid*,*  *Coptis chinensis* Franch |
| 16 | C_17_H_20_O_9_ | 40242-06-6 | 5-O-Feruloylquinic acid | 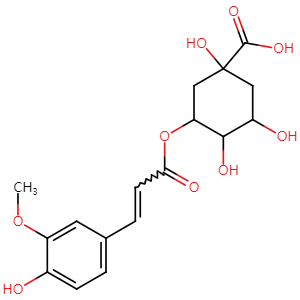 | *Phellodendron chinense* C.K.Schneid |
| 17 | C_23_H_28_O_11_ | 39011-90-0 | Albiflorin | 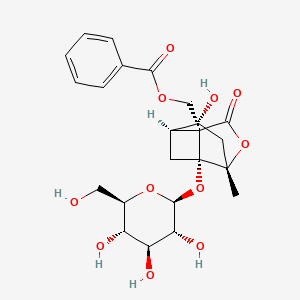 | *Paeonia veitchii* Lynch |
| 18 | C_15_H_14_O_6_ | 490-46-0 | Epicatechin | 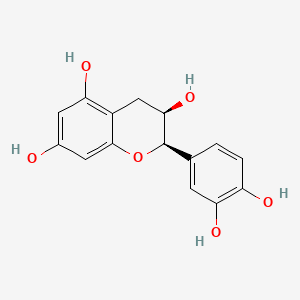 | *Spatholobus suberectus* Dunn |
| 19 | C_23_H_28_O_11_ | 23180-57-6 | Paeoniflorin | 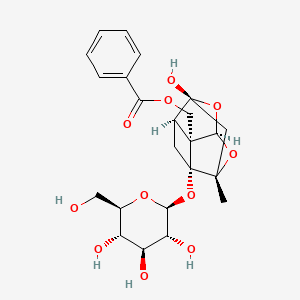 | *Paeonia veitchii* Lynch |
| 20 | C_21_H_25_NO_4_ | 483-14-7 | Tetrahydropalmatine | 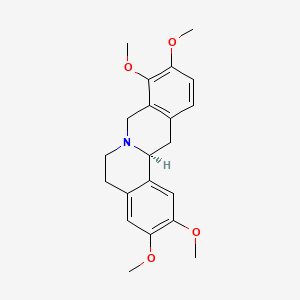 | *Phellodendron chinense* C.K.Schneid*,*  *Coptis chinensis* Franch |
| 21 | C_19_H_16_NO_4_^+^ | 38691-95-1 | Groenlandicine | 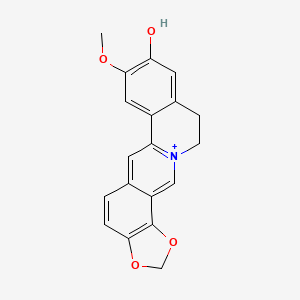 | *Coptis chinensis* Franch |
| 22 | C_20_H_17_NO_5_ | 549-21-3 | Oxyberberine | 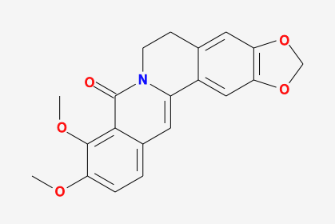 | *Coptis chinensis* Franch |
| 23 | C_17_H_20_O_9_ | 62929-69-5 | 3-O-Feruloylquinic acid | 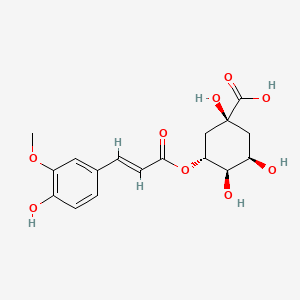 | *Phellodendron chinense* C.K.Schneid |
| 24 | C_19_H_14_NO_4_^+^ | 3486-66-6 | Coptisine | 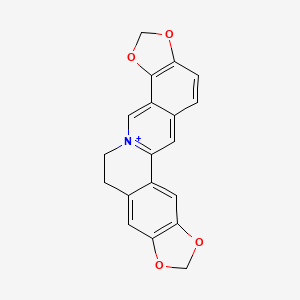 | *Coptis chinensis* Franch*,*  *Phellodendron chinense* C.K.Schneid |
| 25 | C_17_H_20_O_9_ | 2613-86-7 | 4-O-Feruloylquinic acid | 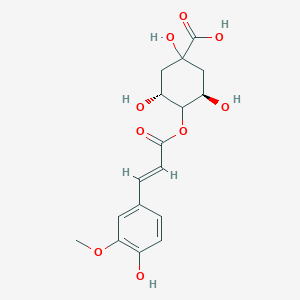 | *Phellodendron chinense* C.K.Schneid |
| 26 | C_20_H_20_NO_4_^+^ | 3621-36-1 | Columbamine | 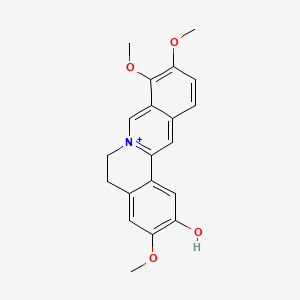 | *Phellodendron chinense* C.K.Schneid*,*  *Coptis chinensis* Franch |
| 27 | C_22_H_22_O_10_ | 20633-67-4 | Calycosin-7-glucoside | 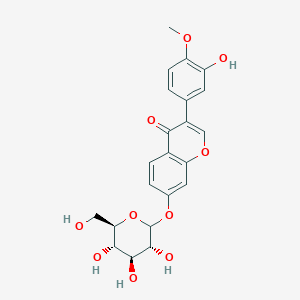 | *Astragalus mongholicus* Bunge |
| 28 | C_20_H_20_NO_4_^+^ | 3621-38-3 | Jatrorrhizine | 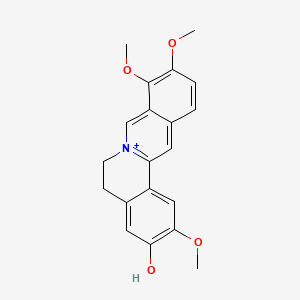 | *Phellodendron chinense* C.K.Schneid*,*  *Coptis chinensis* Franch |
| 29 | C_20_H_22_O_8_ | 27208-80-6 | Polydatin | 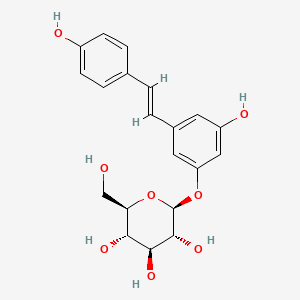 | *Reynoutria japonica* Houtt |
| 30 | C_20_H_18_NO_4_^+^ | 2086-83-1 | Berberine | 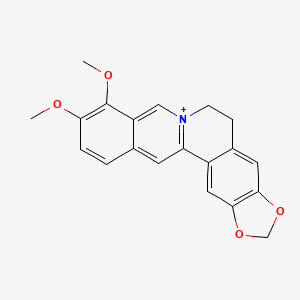 | *Phellodendron chinense* C.K.Schneid*,*  *Coptis chinensis* Franch |
| 31 | C_21_H_22_NO_4_^+^ | 3486-67-7 | Palmatine | 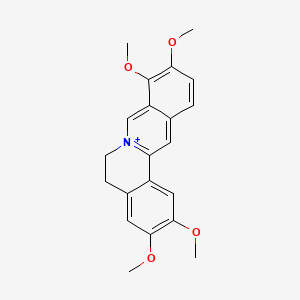 | *Phellodendron chinense* C.K.Schneid*,*  *Coptis chinensis* Franch |
| 32 | C_36_H_66_N_6_O_6_ | NA | Cyclohexa isoleucine Isomer | NA | *Spatholobus suberectus* Dunn |
| 33 | C_42_H_77_N_7_O_7_ | NA | Cycloheptal isoleuine Isomer | NA | *Spatholobus suberectus* Dunn |
| 34 | C_22_H_22_O_9_ | 486-62-4 | Ononin | 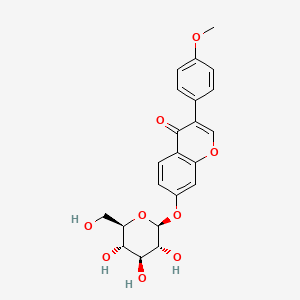 | *Astragalus mongholicus* Bunge*,*  *Spatholobus suberectus* Dunn |
| 35 | C_18_H_16_O_8_ | 20283-92-5 | Rosmarinic acid | 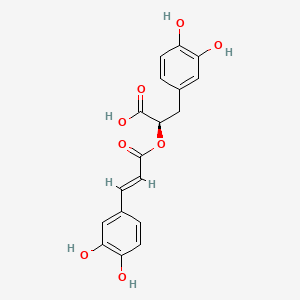 | *Salvia miltiorrhiza* Bunge |
| 36 | C_21_H_20_O_10_ | 33037-46-6 | Aloe-emodin-8-O-β-D-glucoside | 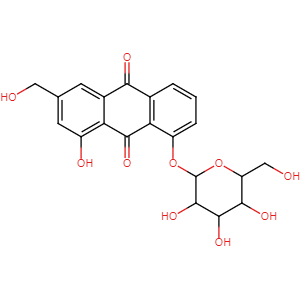 | *Reynoutria japonica* Houtt |
| 37 | C_27_H_22_O_12_ | 28831-65-4 | Lithospermic acid | 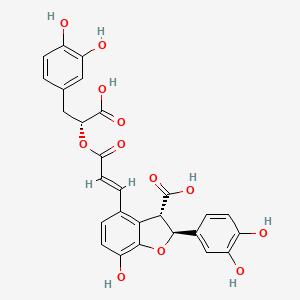 | *Salvia miltiorrhiza* Bunge |
| 38 | C_20_H_24_O_9_ | 64032-49-1 | Torachrysone 8-O-glucoside | 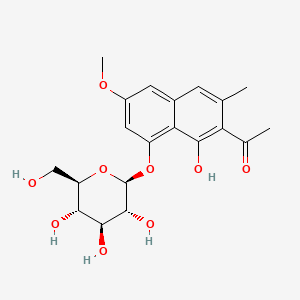 | *Reynoutria japonica* Houtt |
| 39 | C_21_H_20_O_10_ | 23313-21-5 | Emodin 8-O-glucoside | 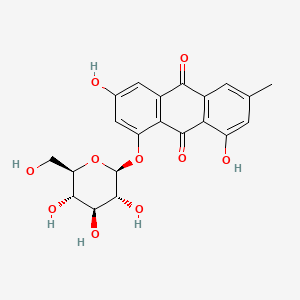 | *Reynoutria japonica* Houtt |
| 40 | C_36_H_30_O_16_ | 121521-90-2 | Salvianolic acid B | 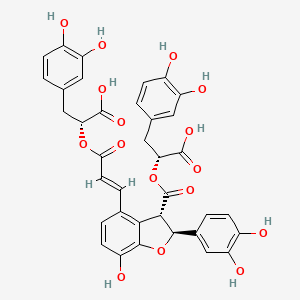 | *Salvia miltiorrhiza* Bunge |
| 41 | C_30_H_32_O_12_ | 38642-49-8 | Benzoylpaeoniflorin | 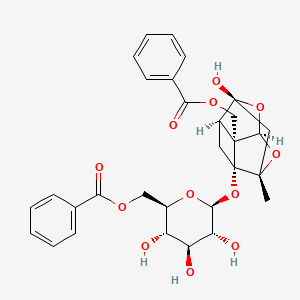 | *Paeonia veitchii* Lynch |
| 42 | C_30_H_32_O_12_ | 184103-78-4 | Benzoylalbiflorin | 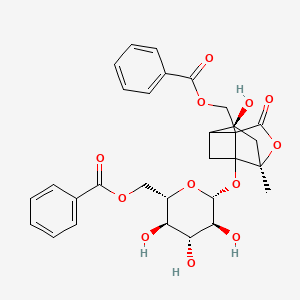 | *Paeonia veitchii* Lynch |
| 43 | C_22_H_26_O_10_ | 1184734-25-5 | Torachrysone O-acetylglucoside | NA | *Reynoutria japonica* Houtt |
| 44 | C_26_H_30_O_8_ | 1180-71-8 | Limonin | 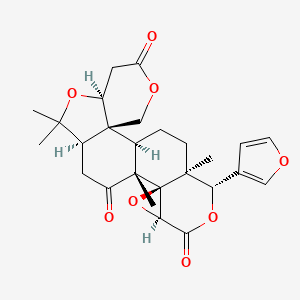 | *Phellodendron chinense* C.K.Schneid*,*  *Coptis chinensis* Franch |
| 45 | C_41_H_68_O_14_ | 84687-43-4 | Astragaloside IV | 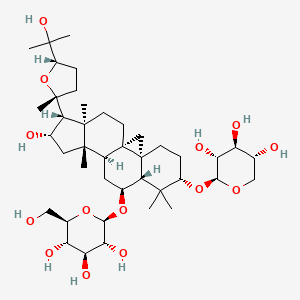 | *Astragalus mongholicus* Bunge |
| 46 | C_41_H_68_O_14_ | 136033-55-1 | Isoastragaloside IV | 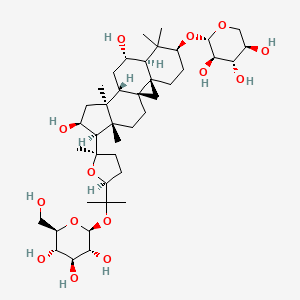 | *Astragalus mongholicus* Bunge |
| 47 | C_43_H_70_O_15_ | 91739-01-4 | Astragaloside II | 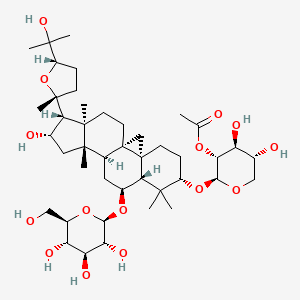 | *Astragalus mongholicus* Bunge |
| 48 | C_43_H_70_O_15_ | 86764-11-6 | Isoastragaloside II | 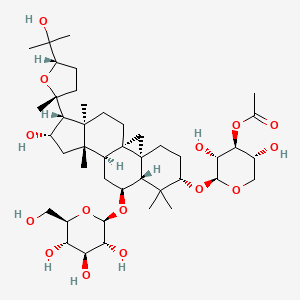 | *Astragalus mongholicus* Bunge |
| 49 | C_45_H_72_O_16_ | 91739-00-3 | Astragaloside I | 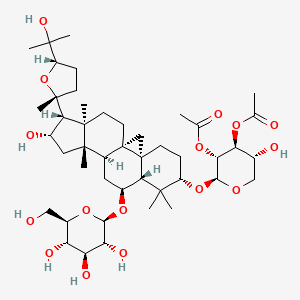 | *Astragalus mongholicus* Bunge |
| 50 | C_19_H_16_O_4_ | 146362-71-2 | Tanshinaldehyde | 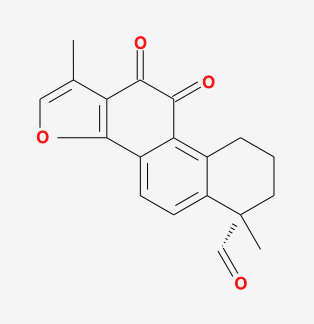 | *Salvia miltiorrhiza* Bunge |
| 51 | C_45_H_72_O_16_ | 84676-88-0 | Isoastragaloside I | 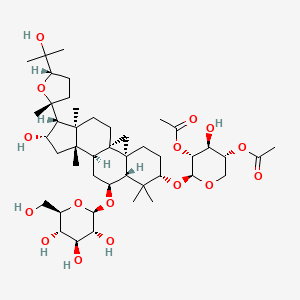 | *Astragalus mongholicus* Bunge |
| 52 | C_15_H_10_O_5_ | 518-82-1 | Emodin | 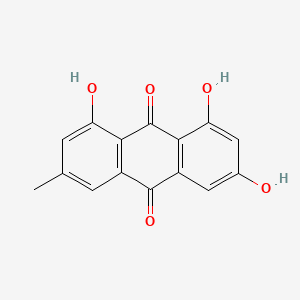 | *Reynoutria japonica* Houtt*,*  *Spatholobus suberectus* Dunn |
| 53 | C_19_H_20_O_3_ | 35825-57-1 | Cryptotanshinone | 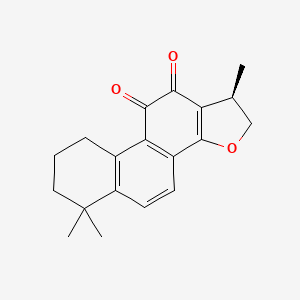 | *Salvia miltiorrhiza* Bunge |
